# *ASXL1* mutation confers poor prognosis in primary myelofibrosis patients with low *JAK2*V617F allele burden but not in those with high allele burden

**DOI:** 10.1038/s41408-020-00364-5

**Published:** 2020-10-12

**Authors:** Yu-Hung Wang, Chien-Chin Lin, Sze-Hwei Lee, Xavier Cheng-Hong Tsai, Shan-Ju Wu, Hsin-An Hou, Tai-Chung Huang, Yuan-Yeh Kuo, Ming Yao, Koping Chang, Chung-Wu Lin, Yun-Chu Lin, Fen-Ming Tien, Wen-Chien Chou, Jih-Luh Tang, Hwei-Fang Tien

**Affiliations:** 1 Graduate Institute of Clinical Medicine, College of Medicine, National Taiwan University, Taipei, Taiwan; 2 Division of Hematology, Department of Internal Medicine, National Taiwan University Hospital, Taipei, Taiwan; 3 Department of Laboratory Medicine, National Taiwan University Hospital, Taipei, Taiwan; 4 Tai-Cheng Stem Cell Therapy Center, National Taiwan University, Taipei, Taiwan; 5 Department of Pathology, National Taiwan University Hospital, Taipei, Taiwan; 6 Department of Hematological Oncology, National Taiwan University Cancer Center, Taipei, Taiwan

**Keywords:** Myeloproliferative disease, Cancer genetics

Dear Editor,

*JAK2*V617F is the most common driver mutation identified in primary myelofibrosis (PMF), followed by *CALR* and *MPL* mutations. In addition to the International Prognostic Scoring System (IPSS), Dynamic IPSS (DIPSS), and DIPSS plus, three widely adopted prognostic systems for PMF patients^1–3^, the prognostic implication of *JAK2*V617F allele burden on the survival of PMF patients has also been evaluated^4–6^. Tefferi et al. first reported that PMF patients with lower *JAK2*V617F allele burden had a poorer prognosis compared to those with higher allele burden, and the observation was verified by Guglielmelli et al.^5^.

Besides, *ASXL1*, *EZH2*, *SRSF2*, and *IDH* mutations were reported to contribute to the disease progression and acute leukemia transformation of PMF, and thus classified as high-molecular risk (HMR) mutations^7,8^. Among them, *ASXL1* mutation is the most frequently harbored mutation.

While the *JAK2*V617F allele burden predicts the prognosis of PMF patients, its clinical association with *ASXL1* mutation remains unexplored. In this study, we found that *ASXL1* mutation confers poor prognosis in PMF patients with low *JAK2*V617F allele burden but not in those with high allele burden, irrespective of DIPSS-plus/DIPSS/IPSS risk classification and other HMR mutations.

We retrospectively enrolled 122 adult patients with PMF diagnosed at the National Taiwan University Hospital (NTUH) from 2005 to 2019. The pathological diagnoses based on the 2016 World Health Organization classifications^9,10^ and fibrosis grading were reviewed by two hematopathologists. A TruSight myeloid sequencing panel and the HiSeq platform were adopted to analyze the gene alterations and mutant allele burden of 54 myeloid-neoplasm relevant genes (Supplementary Table 1) on bone marrow or whole blood cells obtained at the time of PMF diagnosis or referral. This study was approved by the Research Ethics Committee of NTUH (Project number: 201709072RINC).

The median age of the 122 PMF patients was 61 years. Thirteen patients had pre-PMF, whereas 109 patients had overt PMF. The clinical and laboratory characteristics of these patients at diagnosis or first referral are shown in Supplemental Table 2. *JAK2*V617F was the most common driver mutation (64.8%), followed by *CALR* (18%) and *MPL* mutations (9%).

The mutational landscape of 122 PMF patients is illustrated in Supplemental Fig. 1. *ASXL1* mutation was the most common (37%) mutation other than driver mutations, followed by *TET2* (16%) and *EZH2* mutations (12%). Overall, 88 (72%) patients harbored at least one additional mutation; 33, 16, 10, and 13% of the patients harbored 1, 2, 3, or 4 or more mutations other than *JAK2*/*CALR*/*MPL*, respectively. Fifty-two patients (42.6%) had at least one HMR mutation (*ASXL1*, *EZH2*, *SRSF2*, and *IDH* mutations). Details of mutation status and allele burden are shown in Supplemental Table 3.

With a median follow-up time of 28.2 months, the median overall survival (OS) of all cohort was not reached (NR) and was not different among patients with different driver mutations (*p* = 0.342, Supplementary Fig. 2a). Meanwhile, patients with type 1/like *CALR* mutation tended to have a better OS than those with type 2/like *CALR* mutations (*p* = 0.051, Supplementary Fig. 2b). Patients with pre-PMF and overt-PMF had significantly different leukemia-free survival (LFS) and OS (Supplementary Fig. 3a and b, respectively). Moreover, patients with at least one HMR mutation had significantly shorter LFS and OS than those without (Supplementary Fig. 3c and d, respectively). Conceivably, patients harboring more HMR mutations had worse LFS and OS (Supplementary Fig. 3e and f, respectively). The patients’ OS was well risk-stratified by prognostic scoring systems, including IPSS, DIPSS plus, MIPSS70, and GIPSS (Supplementary Fig. 4 and Supplemental Table 4).

We divided the 79 patients who had *JAK2*V617F and available data of allele burden into low- and high-allele burden groups using 75%, the median, as the cutoff. Concurring with previous studies, patients with lower *JAK2*V617F allele burden had significantly inferior OS than those with higher burden (*p* = 0.031, Fig. 1a). Among the *JAK2*-mutated patients, those with *ASXL1* mutation had a significantly shorter OS than those without (*p* = 0.012, Fig. 1b). Taken *ASXL1* mutation and allele burden of *JAK2*V617F together, the mutation of *ASXL1* conferred worse OS in those with lower *JAK2*V617F allele burden but not in those with higher allele burden (*p* < 0.001 and *p* = 0.703, Supplemental Fig. 5). By and large, patients with concurrent *ASXL1* mutation and low *JAK2*V617F allele burden had a distinctively worse survival than those with other three mutation combinations (*p* < 0.001, Fig. 1c). Besides, this group of patients also had the shortest survival among the total cohort with *JAK2*-wild patients included (*p* < 0.001, Supplementary Fig. 6). We further levelled off the cutoff value of allele burden to 50%, approximately the lower quadrant of this cohort. Consistently, *ASXL1* mutation conferred poor prognosis in those with lower *JAK2*V617F allele burden but not in those with higher allele burden (Supplementary Fig. 7).

**Fig. 1:**
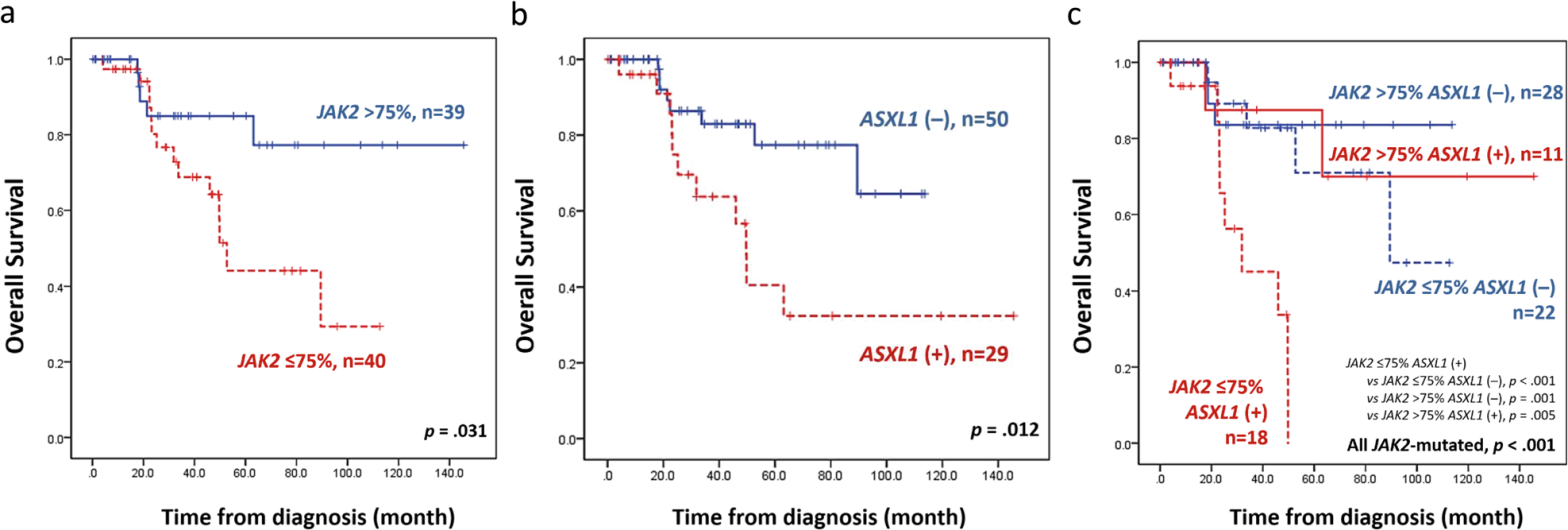
Kaplan–Meier survival curves of 79 *JAK2*-mutated PMF patients. **a** OS stratified by *JAK2* allele burden. Patients with lower *JAK2* allele burden had inferior survival. **b** OS stratified by mutation status of *ASXL1* among *JAK2*-mutated patients. Patients with concurrent *ASXL1* mutation had inferior survival. **c** OS stratified by combined *JAK2* allele burden and *ASXL1* mutation status. Patients with concurrent *ASXL1* mutation and low *JAK2* allele burden had distinctively inferior survival.

In light of this interesting finding, we compared the clinical and laboratory characteristics between patients with higher and lower *JAK2*V617F allele burden. Nevertheless, there was no significant difference between groups, except that patients with lower *JAK2* allele burden more frequently harbored *SRSF2* mutation (Supplementary Table 5). The mutation details of *ASXL1* in *JAK2*-mutated patients are displayed in Supplementary Table 6.

In multivariable analysis, we used DIPSS-plus, *ASXL1* mutation/*JAK2*V617F allele burden, and mutation statuses of *EZH2*, *SRSF2*, and *IDH* as variables. *SETBP1*, *ETV6*, and *KDM6A* mutations were not included due to low prevalence in this cohort despite of their prognostic impact in univariate analysis (Supplemental Table 7). Mutation of *ASXL1* along with low allele burden of *JAK2*V617F appeared as an independent adverse risk factor for both LFS and OS (hazard ratio, HR: 2.061, *p* = 0.045; and HR: 2.361, *p* = 0.012, respectively, Table 1), irrespective of DIPSS-plus risk groups and mutation statuses of other HMR genes. The prognostic significance of combined *ASXL1* mutation and low *JAK2*V617F allele burden remained valid for OS in the analysis applying IPSS and DIPSS, respectively (Supplementary Tables 8 and 9).

**Table 1:** Multivariable analysis for LFS and OS in the 79 *JAK2*-mutated PMF patients, adopting DIPSS-plus, *ASXL1* mutation and *JAK2* allele burden, and other HMR gene mutations as variables.

	LFS 95% CI				OS 95% CI			
Variable	HR	Lower	Upper	*P*	HR	Lower	Upper	*P*
DIPSS-plus^a^	1.984	0.981	4.012	0.057	2.034	0.983	4.212	0.056
*ASXL1*/*JAK2* allele burden^b^	2.061	1.015	4.186	0.045	2.361	1.206	4.624	0.012
*EZH2*	1.932	0.456	8.181	0.371	1.567	0.384	6.388	0.531
*SRSF2*	2.074	0.414	10.394	0.375	1.504	0.324	6.979	0.602
*IDH*	2.319	0.079	68.062	0.626	4.134	0.152	112.1	0.399

*P* values < 0.05 are considered statistically significant.

*HMR* high-molecular risk, *HR* hazard ratios, *CI* confidence interval.

^a^DIPSS-plus: low vs. intermediate-1 vs. intermediate-2 vs. high-risk groups.

^b^*ASXL1* mutation with low *JAK2* allele burden (<75%) versus others.

Since the survival of PMF patients may vary from months to more than a decade, the identification of prognostic factors has been of great interest to physicians and scientists. Tefferi et al. first described the impact of *JAK2*V617F allele burden on PMF patients’ survival: patients with *JAK2*V617F allele burden in the lower quartile had significantly reduced OS, compared with those with allele burden in middle or upper quartile, or those without a mutant *JAK2*^4^. This finding was supported by the study of Guglielmelli et al.^5^, within which a low *JAK2*V617F allele burden was confirmed as an independent adverse risk factor^5^.

Although the pathophysiologic mechanism underlying this correlation and the optimal cutoff value remain unknown, the contention that a lower *JAK2*V617F allele burden at diagnosis is associated with inferior survival in PMF patients is widely accepted. On the other hand, the role of a higher *JAK2*V617F allele burden in the MF phenotype is yet to be established. In this cohort, patients with higher and lower allele burden did not differ in clinical and laboratory features, except for a higher incidence of *SRSF2* mutation in the latter group.

The *ASXL1* mutations are correlated with adverse prognosis in PMF^5^. In a study of Guglielmelli et al.^7^, mutant *ASXL1* and other three mutations (*EZH2*, *SRSF2*, and *IDH1*/*2* mutations) were categorized as high-molecular risk mutations due to their detrimental effects on PMF patients’ survival. Recently, Tefferi et al identified seven adverse mutations by targeted deep sequencing and multivariable analysis^8^. Among them, *ASXL1* mutation was the most frequently detected and significantly associated with inferior survival.

In this study, we analyzed the combined effect of *JAK2*V617F allele burden and *ASXL1* mutation on the survival of PMF patients. We identified a distinct patient population, characterized by having concurrent *ASXL1* mutation and low *JAK2*V617F allele burden, who had a significantly shorter OS than others not only in patients with *JAK2*V617F, but also in the total cohort. The prognostic impact of combined *ASXL1* mutation and low allele burden of *JAK2*V617F remained significant in multivariable analysis for OS. As hematopoietic stem cell transplantation (HSCT) is currently the only curative treatment for PMF, the optimal timing and selection of candidates for transplant remain to be defined^11–13^. The International Working Group-Myeloproliferative Neoplasms Research and Treatment and European LeukemiaNet suggest that patients with intermediate-1 risk may consider HSCT if they harbor *ASXL1* mutation^14^. However, in clinical practice, physicians may not optimally adhere to the guideline due to the risks HSCT introduces. Incorporation of the *JAK2*V617F allele burden to current risk stratification might provide complementary information to treatment decisions in the future^11^.

This study was limited by its retrospective nature; thereby diverse confounding factors might become difficult to assess. Additionally, while most specimens were obtained at the time of diagnosis, a small proportion of patients’ samples were drawn at their referral to NTUH. As the allele burden of *JAK2*V617F is known to evolve throughout disease and treatment, the allele burden assessment could be, therefore, confounded. Despite of these, we have demonstrated that *ASXL1* mutation accompanied by a low *JAK2*V617F allele burden dictated a distinct patient population with significantly reduced survival, and its independent prognostic relevance was validated in multivariable analysis. Prospective and experimental studies are warranted to support these observations and ascertain the underlying mechanisms.

## Supplementary information

Supplemental material

## Data Availability

The data reported in this article could be accessed through 10.6084/m9.figshare.12952022 once the manuscript is published, or request with the corresponding author.
