# Supplementary material for: ASXL1 mutation confers poor prognosis in primary myelofibrosis patients with low JAK2V617F allele burden but not in those with high allele burden
Source: Blood Cancer J. 2020 Oct 12;10(10):99. doi: 10.1038/s41408-020-00364-5 (PMC7550588; doi:10.1038/s41408-020-00364-5)
Supplement: Supplementary file 1 — Supplemental material [file 41408_2020_364_MOESM1_ESM.docx]

**Supplemental Table 1. Full list of 54 myeloid neoplasm-relevant genes studied in targeted NGS sequencing.**

| **Gene name** | **Target region (exon)** | **Gene name** | **Target region (exon)** |
| --- | --- | --- | --- |
| ***ABL*** | 4-6 | ***JAK3*** | 13 |
| ***ASXL1*** | 12 | ***KDM6A*** | full |
| ***ATRX*** | 8-10 and 17-31 | ***KIT*** | 2, 8-11, 13+17 |
| ***BCOR*** | full | ***KRAS*** | 2+3 |
| ***BCORL1*** | full | ***MLL*** | 5-8 |
| ***BRAF*** | 15 | ***MPL*** | 10 |
| ***CALR*** | 9 | ***MYD88*** | 3-5 |
| ***CBL*** | 8+9 | ***NOTCH1*** | 26-28, 34 |
| ***CBLB*** | 9, 10 | ***NPM1*** | 12 |
| ***CBLC*** | 9, 10 | ***NRAS*** | 2+3 |
| ***CDKN2A*** | full | ***PDGFRA*** | 12, 14, 18 |
| ***CEBPA*** | full | ***PHF6*** | full |
| ***CSF3R*** | 14-17 | ***PTEN*** | 5+7 |
| ***CUX1*** | full | ***PTPN11*** | 3+13 |
| ***DNMT3A*** | full | ***RAD21*** | full |
| ***ETV6*** | full | ***RUNX1*** | full |
| ***EZH2*** | full | ***SETBP1*** | 4 (partial) |
| ***FBXW7*** | 9+10+11 | ***SF3B1*** | 13-16 |
| ***FLT3*** | 14+15+20 | ***SMC1A*** | 2, 11, 16+17 |
| ***GATA1*** | 2 | ***SMC3*** | 10, 13, 19, 23, 25+28 |
| ***GATA2*** | 2-6 | ***SRSF2*** | 1 |
| ***GNAS*** | 8+9 | ***STAG2*** | full |
| ***HRAS*** | 2+3 | ***TET2*** | 3-11 |
| ***IDH1*** | 4 | ***TP53*** | 2-11 |
| ***IDH2*** | 4 | ***U2AF1*** | 2+6 |
| ***IKZF1*** | full | ***WT1*** | 7+9 |
| ***JAK2*** | 12+14 | ***ZRSR2*** | full |

**Supplemental Table 2. Comparison of clinical and laboratory features between patients with pre- and overt primary myelofibrosis**

| **Clinical characters** | **Total (N=122)** | **Pre-PMF (n=13)** | **Overt PMF (n=109)** | ***P* value** |
| --- | --- | --- | --- | --- |
| **Male** | 68 (55.7) | 8 (61.5) | 60 (55) | 0.772 |
| **Age*** | 61 (21-88) | 62 (34-76) | 61 (21-88) | 0.950 |
| **Laboratory data*** |  |  |  |  |
| **WBC, X 10^9^ /L** | 14.1 (0.6-27.3) | 10.3 (6.4-23.3) | 14.3 (0.6-27.3) | 0.557 |
| **Hb, g/dL** | 10.1 (3.9-18.3) | 13.4 (9.2-16.1) | 9.8 (3.9-18.3) | 0.003 |
| **Platelet, X 10^9^ /L** | 341 (7-1378) | 641 (325-1378) | 318 (7-270) | 0.017 |
| **Circulating blast, X 10^9^ /L** | 0.5 (0-7) | 0 | 1 (0-7) | <0.001 |
| **Driver mutations†, n(%)** |  |  |  | 0.987 |
| ***JAK2*** | 79 (64.8) | 9 (69.2) | 70 (64.2) |  |
| ***CALR*** | 22 (18) | 2 (15.4) | 20 (18.3) |  |
| **Type 1-like** | 16 | 2 (100) | 14 (70) |  |
| **Type 2-like** | 6 | 0 (0) | 6 (30) |  |
| ***MPL*** | 11 (9) | 1 (7.7) | 10 (9.2) |  |
| **Triple-negative** | 10 (8.2) | 1 (7.7) | 9 (8.3) |  |
| **Unfavorable cytogenetics‡, n (%)** | 7 (6.8) | 1 (11.1) | 6 (6.4) | 0.483 |
| **IPSS, n (%)** |  |  |  | <0.001 |
| **Low / intermediate-1** | 32 (26.2) | 10 (76.9) | 22 (20.2) |  |
| **Intermediate-2 / High** | 90 (72.8) | 3 (23.1) | 87 (79.8) |  |
| **DIPSS, n (%)** |  |  |  | <0.001 |
| **Low / intermediate-1** | 46 (37.7) | 11 (84.6) | 35 (32.1) |  |
| **Intermediate-2 / High** | 76 (62.3) | 2 (15.4) | 74 (67.9) |  |
| **High molecular risk mutation, n (%)** | 52 (42.6) | 2 (15.4) | 50 (45.9) | 0.041 |
| **Acute transformation, n (%)** | 11 (9) | 0 (0) | 11 (10.1) | 0.605 |
| **Treatment, n (%)** |  |  |  |  |
| **Hydroxyurea** | 63 (52.1) | 7 (53.8) | 56 (51.9) | >0.999 |
| ***JAK2* inhibitor** | 46 (38) | 3 (23.1) | 43 (39.8) | 0.366 |
| **Anagrelide** | 35 (28.9) | 6 (46.2) | 29 (26.9) | 0.195 |
| **Splenectomy/spleen radiation** | 21 (19.8) | 0 (0) | 21 (22.1) | 0.116 |
| **Allo-HSCT** | 17 | 0 (0) | 17 (15.6) | 0.211 |

*P* values < .05 are considered statistically significant.

*median (range)

†Thirteen patients with pre-PMF and 109 patients with overt PMF had next-generation sequencing data.

‡Unfavorable cytogenetics: complex karyotype (≧3 changes), +8, −7/7q−, i(17q), inv(3), −5/5q−, 12p− or 11q23 rearrangement

Abbreviations: Allo-HSCT, allogeneic-hematopoietic stem cell transplant.

**Supplemental Table 3. Genetic alterations in 122 PMF patients**

| Genes | Total | Allele burden (%)* | Number of patients with the mutation (%) | | | *P* value |
| --- | --- | --- | --- | --- | --- | --- |
|  |  |  | **Pre-PMF (n=13)** | **Overt PMF (n=109)** | |  |
| *JAK2* | 79 (65.6) | 75 (10.9-97.6) | 9 (66.7) | | 70 (65.4) | >0.999 |
| *CALR* | 22 (18.0) | 53.1 (19.1-70.1) | 3 (20.0) | | 19 (17.8) | 0.733 |
| *MPL* | 11 (9.0) | 70.9 (16-97) | 1 (6.7) | | 10 (9.3) | >0.999 |
| *ASXL1* | 45 (36.9) | 35.7 (5-60) | 1 (6.7) | | 44 (41.1) | 0.009 |
| *EZH2* | 15 (12.3) | 43.4 (6.4-95.6) | 0 (0) | | 15 (14.2) | 0.211 |
| *SRSF2* | 11 (9.0) | 42.8 (7-56) | 1 (6.7) | | 10 (9.3) | >0.999 |
| *IDH1* | 2 (1.6) | 47.7 (46-49) | 1 (6.7) | | 1 (0.9) | 0.232 |
| *IDH2*† | 1 (0.8) | - | 0 (0) | | 1 (0.9) | >0.999 |
| *TET2* | 19 (15.6) | 45.6 (5.4-73.6) | 2 (13.3) | | 17 (15.9) | >0.999 |
| *DNMT3A* | 12 (9.8) | 31.5 (5.4-49) | 2 (13.3) | | 10 (9.3) | 0.642 |
| *TP53* | 8 (6.6) | 50.9 (12-54) | 0 (0) | | 8 (7.5) | 0.594 |
| *CUX1* | 7 (5.7) | 42.4 (8.7-88.5) | 0 (0) | | 7 (6.5) | 0.595 |
| *SETBP1* | 7 (5.7) | 38.8 (12-50) | 0 (0) | | 7 (6.5) | 0.595 |
| *BCOR* | 6 (4.9) | 45.8 (5-99) | 1 (6.7) | | 5 (4.7) | >0.999 |
| *U2AF1* | 6 (4.9) | 43.3 (40-51) | 0 (0) | | 6 (5.6) | >0.999 |
| *NRAS* | 5 (4.1) | 33.8 (8-50) | 0 (0) | | 5 (4.7) | >0.999 |
| *RUNX1* | 5 (4.1) | 43.9 (6-49) | 1 (6.7) | | 4 (3.7) | 0.487 |
| *SF3B1* | 5 (4.1) | 45.5 (6-48) | 0 (0) | | 5 (4.7) | >0.999 |
| *BCORL1* | 4 (3.3) | 48.4 (37-92) | 0 (0) | | 4 (3.7) | >0.999 |
| *STAG2* | 4 (3.3) | 70.2 (26-99) | 0 (0) | | 4 (3.7) | >0.999 |
| *ETV6* | 3 (2.5) | 30.8 (27-45) | 0 (0) | | 3 (2.8) | >0.999 |
| *GATA2* | 3 (2.5) | 8.6 (5-52) | 0 (0) | | 3 (2.8) | >0.999 |
| *GNAS* | 3 (2.5) | 37.8 (32-44) | 0 (0) | | 3 (2.8) | >0.999 |
| *KRAS* | 3 (2.5) | 35.7 (9.8-50.6) | 0 (0) | | 3 (2.8) | >0.999 |
| *PHF6* | 3 (2.5) | 41 (37-59) | 0 (0) | | 3 (2.8) | >0.999 |
| *NOTCH1* | 2 (1.6) | 28.3 (6-50) | 0 (0) | | 2 (1.9) | >0.999 |
| *FBXW72*† | 1 (0.8) | - | 0 (0) | | 1 (0.9) | >0.999 |
| *GATA1*† | 1 (0.8) | - | 0 (0) | | 1 (0.9) | >0.999 |
| *IKZF1*† | 1 (0.8) | - | 0 (0) | | 1 (0.9) | >0.999 |
| *KDM6A*† | 1 (0.8) | - | 0 (0) | | 1 (0.9) | >0.999 |
| *ZRSR2*† | 1 (0.8) | - | 0 (0) | | 1 (0.9) | >0.999 |

*P* values < .05 are considered statistically significant.

*median (range)

†median uncountable due to constant value

**Supplemental Table 4. Patient number and median OS in each risk group**

| System | Risk | Patient number | Median OS (months) |
| --- | --- | --- | --- |
|  |  |  |  |
| IPSS | Low | 11 | Not reached |
|  | Intermediate-1 | 20 | Not reached |
|  | Intermediate-2 | 40 | Not reached |
|  | High | 51 | 49.8 |
| DIPSS | Low | 11 | Not reached |
|  | Intermediate-1 | 35 | Not reached |
|  | Intermediate-2 | 53 | 89.5 |
|  | High | 23 | 49.8 |
| DIPSS plus | Low | 11 | Not reached |
|  | Intermediate-1 | 29 | Not reached |
|  | Intermediate-2 | 57 | 63.1 |
|  | High | 25 | 25.2 |
| MIPSS 70 | Low | 3 | Not reached |
|  | Intermediate | 60 | Not reached |
|  | High | 59 | 33.7 |
| MIPSS 70 plus 2.0 | Low | 2 | Not reached |
|  | Intermediate | 9 | Not reached |
|  | High | 87 | Not reached |
|  | Very high | 24 | 25.2 |
| GIPSS | Low | 7 | Not reached |
|  | Intermediate-1 | 66 | Not reached |
|  | Intermediate-2 | 39 | 32.1 |
|  | High | 10 | 31.7 |

**Supplemental Table 5. Comparison of clinical and laboratory features between *JAK2*-mutated PMF patients with lower- and higher-*JAK2* allele burden**

| **Clinical characters** | **Low *JAK2* (n=40)** | **High *JAK2* (n=39)** | ***P* value** |
| --- | --- | --- | --- |
| **Male** | 26 (65) | 19 (48.7) | 0.176 |
| **Age*** | 63.8 (34-80) | 61.7 (28-87) | 0.430 |
| **Laboratory data*** |  |  |  |
| **WBC, X 10^9^ /L** | 11.4 (1.99-27.3) | 20.9 (0.65-42.9) | 0.056 |
| **Hb, g/dL** | 11.3 (3.9-15) | 11 (6.5-18.3) | 0.912 |
| **Platelet, X 10^9^ /L** | 356 (15-1358) | 349 (18-1640) | 0.912 |
| **Circulating blast, X 10^9^ /L** | 0.25 (0-6) | 0.5 (0-22) | 0.920 |
| **Constitutional symptoms** | 12 (57.1) | 16 (69.6) | 0.533 |
| **Thromboembolic event** | 9 (22.5) | 5 (13.2) | 0.379 |
| **Unfavorable cytogenetics**† | 1 (2.5) | 1 (2.6) | >0.999 |
| **Overt-PMF** | 33 (82.5) | 36 (92.3) | 0.311 |
| **Acute transformation** | 4 (10) | 2 (5.1) | 0.675 |
| **IPSS**‡ |  |  | >0.999 |
| **Low / intermediate-1** | 11 (27.5) | 11 (28.2) |  |
| **Intermediate-2 / High** | 29 (72.5) | 28 (71.8) |  |
| **DIPSS** |  |  | 0.652 |
| **Low / intermediate-1** | 16 (40) | 18 (46.2) |  |
| **Intermediate-2 / High** | 24 (60) | 21 (53.8) |  |
| **High molecular risk mutation** |  |  |  |
| ***ASXL1*** | 18 (45.0) | 11 (28.2) | 0.162 |
| ***EZH2*** | 4 (10.0) | 3 (7.9) | >0.999 |
| ***SRSF2*** | 7 (17.5) | 0 (0) | 0.012 |
| ***IDH*** | 2 (5.0) | 0 (0) | 0.494 |

*P* values < .05 are considered statistically significant.

*median (range)

†Unfavorable cytogenetics: complex karyotype (≧3 changes), +8, −7/7q−, i(17q), inv(3), −5/5q−, 12p− or 11q23 rearrangement

**Supplemental Table 6. Mutation patterns of *ASXL1* in patients with higher- and lower-*JAK2* allele burden**

| **Patient** | ***JAK2* allele burden** | ***ASXL1* mutation** |
| --- | --- | --- |
| 15 | High | p.Gly646TrpfsTer12 |
| 26 | High | p.Glu635ArgfsTer15 |
| 35 | High | p.Trp796GlyfsTer22 |
| 38 | High | p.Gly646TrpfsTer12 |
| 50 | High | p.Gly869ValfsTer7 |
| 69 | High | p.Gly646TrpfsTer12 |
| 84 | High | p.Arg693Ter |
| 108 | High | p.Tyr591Ter |
| 115 | High | p.Arg693Ter |
| 121 | High | p.R693X |
| 122 | High | p.G646Wfs*12 |
| 5 | Low | p.Gly646TrpfsTer12 |
| 6 | Low | p.Leu775Ter |
| 8 | Low | p.Gly646TrpfsTer12 |
| 13 | Low | p.Arg965Ter |
| 19 | Low | p.Ile593Val |
| 20 | Low | p.Asp954Ter |
| 27 | Low | p.Gly679Ter |
| 34 | Low | p.Glu635ArgfsTer15 |
| 60 | Low | p.Arg620CysfsTer14 |
| 65 | Low | p.Gly646TrpfsTer12 |
| 66 | Low | p.Arg693Ter |
| 72 | Low | p.Cys605LeufsTer14 |
| 90 | Low | p.Gly646TrpfsTer12 |
| 91 | Low | p.Leu775Ter |
| 94 | Low | p.Gly646TrpfsTer12 |
| 96 | Low | p.Gly643GlufsTer15 |
| 102 | Low | p.Gly646TrpfsTer12 |
| 110 | Low | p.Pro808LeufsTer10 |

**Supplemental Table 7. Univariate analysis (Cox regression) of the impact of different variables on the overall survival in the 122 PMF patients**

| **Variable** | **HR** | **Lower 95% CI** | **Upper 95% CI** | ***P* value** |
| --- | --- | --- | --- | --- |
| **Age*** | 1.042 | 1.015 | 1.069 | 0.002 |
| **Lower *JAK2* allele burden** | 2.956 | 1.056 | 8.278 | 0.039 |
| **Mutation numbers*^,^**† | 1.571 | 1.275 | 1.934 | <0.001 |
| ***ASXL1*** | 5.024 | 2.338 | 10.795 | <0.001 |
| ***EZH2*** | 6.239 | 2.827 | 13.768 | <0.001 |
| ***SRSF2*** | 6.832 | 2.699 | 17.296 | <0.001 |
| ***IDH1/2*** | 6.097 | 0.792 | 46.956 | 0.083 |
| ***TET2*** | 0.508 | 0.153 | 1.686 | 0.268 |
| ***DNMT3A*** | 0.292 | 0.040 | 2.150 | 0.227 |
| ***TP53*** | 1.595 | 0.477 | 5.331 | 0.448 |
| ***CUX1*** | 0.471 | 0.064 | 3.465 | 0.459 |
| ***SETBP1*** | 6.870 | 2.778 | 16.988 | <0.001 |
| ***BCOR*** | 2.226 | 0.662 | 7.481 | 0.196 |
| ***U2AF1*** | 2.747 | 0.829 | 9.105 | 0.098 |
| ***NRAS*** | 2.514 | 0.761 | 8.307 | 0.131 |
| ***RUNX1*** | 1.596 | 0.379 | 6.721 | 0.524 |
| ***SF3B1*** | 1.827 | 0.433 | 7.704 | 0.412 |
| ***BCORL1*** | 2.669 | 0.351 | 20.297 | 0.343 |
| ***STAG2*** | 1.794 | 0.423 | 7.600 | 0.428 |
| ***ETV6*** | 14.591 | 1.583 | 134.524 | 0.018 |
| ***GATA2*** | 3.593 | 0.839 | 15.399 | 0.085 |
| ***GNAS*** | 0.048 | <0.001 | 3108.402 | 0.590 |
| ***KRAS*** | 1.534 | 0.206 | 11.404 | 0.676 |
| ***PHF6*** | 1.623 | 0.219 | 12.037 | 0.636 |
| ***NOTCH1*** | 0.047 | <0.001 | 468.564 | 0.515 |
| ***FBXW72*** | 0.049 | <0.001 | 383350.2 | 0.709 |
| ***GATA1*** | 0.048 | <0.001 | 3624.404 | 0.595 |
| ***IKZF1*** | 0.049 | <0.001 | 1.729 E+40 | 0.951 |
| ***KDM6A*** | 10.848 | 1.354 | 86.905 | 0.025 |
| ***ZRSR2*** | 2.460 | 0.332 | 18.236 | 0.379 |

*P* values < .05 are considered statistically significant.

Abbreviations: HR, hazard ratios; CI, confidence interval.

*as continuous variable

†mutation numbers besides of driver mutations

**Supplemental Table 8. Multivariable analysis for LFS and OS in the 79 *JAK2*-mutated PMF patients, adopting IPSS, *ASXL1* mutation and *JAK2* allele burden, and other HMR gene mutations as variables**

|  | LFS | | | | OS | | | |  |
| --- | --- | --- | --- | --- | --- | --- | --- | --- | --- |
|  | 95% CI | | | | 95% CI | | | |  |
| Variable | **HR** | **Lower** | **Upper** | ***P*** | **HR** | **Lower** | **Upper** | ***P*** | |
| IPSS* | 2.662 | 1.166 | 6.076 | 0.020 | 2.511 | 1.079 | 5.843 | 0.033 | |
| *ASXL1*/*JAK2* allele burden† | 1.778 | 0.897 | 3.524 | 0.099 | 2.036 | 1.054 | 3.935 | 0.034 | |
| *EZH2* | 1.558 | 0.388 | 6.262 | 0.532 | 1.315 | 0.335 | 5.162 | 0.695 | |
| *SRSF2* | 3.564 | 0.740 | 17.170 | 0.113 | 2.613 | 0.579 | 11.801 | 0.212 | |
| *IDH* | 1.789 | 0.069 | 46.068 | 0.726 | 3.164 | 0.128 | 78.382 | 0.482 | |

*P* values < .05 are considered statistically significant.

Abbreviations: HR, hazard ratios; CI, confidence interval.

*IPSS: low vs. intermediate-1 vs. intermediate-2 vs. high-risk groups

†*ASXL1* mutation with low *JAK2* allele burden versus others.

**Supplemental Table 9. Multivariable analysis for LFS and OS in the 79 *JAK2*-mutated PMF patients, adopting DIPSS, *ASXL1* mutation and *JAK2* allele burden, and other HMR gene mutations as variables**

|  | LFS | | | | OS | | | |  |
| --- | --- | --- | --- | --- | --- | --- | --- | --- | --- |
|  | 95% CI | | | | 95% CI | | | |  |
| Variable | **HR** | **Lower** | **Upper** | ***P*** | **HR** | **Lower** | **Upper** | ***P*** | |
| DIPSS* | 1.957 | 0.976 | 3.922 | 0.059 | 1.790 | 0.862 | 3.719 | 0.119 | |
| *ASXL1*/*JAK2* allele burden† | 2.096 | 1.045 | 4.201 | 0.037 | 2.429 | 1.253 | 4.709 | 0.009 | |
| *EZH2* | 1.067 | 0.219 | 5.189 | 0.936 | 0.941 | 0.195 | 4.550 | 0.940 | |
| *SRSF2* | 1.878 | 0.367 | 9.614 | 0.450 | 1.439 | 0.301 | 6.881 | 0.649 | |
| *IDH* | 4.004 | 0.147 | 109.34 | 0.411 | 7.093 | 0.278 | 181.11 | 0.236 | |

*P* values < .05 are considered statistically significant.

Abbreviations: HR, hazard ratios; CI, confidence interval.

*DIPSS: low vs. intermediate-1 vs. intermediate-2 vs. high-risk groups

†*ASXL1* mutation with low *JAK2* allele burden versus others.

**Supplemental Figure legends**

**Supplemental Figure 1. Mutational landscape of 122 PMF patients.**

**Supplemental Figure 2. Kaplan-Meier survival curves of 122 PMF patients with different driver mutations**

(a) OS of 122 PMF patients stratified by different driver mutations. No significant difference was found among groups. (b) OS of 22 *CALR*-mutated PMF patients. Patients with type 1/like *CALR* mutation tended to have a better OS than those with type 2/like *CALR* mutations.

**Supplemental Figure 3. Kaplan-Meier survival curves of PMF patients stratified by the status of the disease and HMR mutations**

(a) and (b) LFS and OS of the 122 PMF patients in different disease statuses. Patients with pre-PMF had better LFS and OS than those with overt-PMF. (c) and (d) LFS and OS of the 122 patients with or without high molecular risk (HMR) genes (*ASXL1*, *EZH2*, *SRSF2*, and *IDH1*/*2*). Patients with HMR mutations had inferior LFS and OS. (e) and (f) LFS and OS of the 122 patients harboring 0, 1 or 2 or more HMR genes. Patients with more HMR genes had inferior LFS and OS.

**Supplemental Figure 4. Kaplan-Meier survival curves stratified by different scoring systems.**

OS of 122 PMF patients stratified by (a) IPSS, (b) DIPSS-plus, (c) MIPSS70 and (d) GIPSS.

**Supplemental Figure 5. Kaplan-Meier survival curves of *JAK2*-mutated PMF patients with allele burden lower or higher than 75% stratified by the status of *ASXL1* mutation.**

(a) OS of 40 patients with *JAK2* allele burden lower than 75%. Patients with *ASXL1* mutation had significantly shorter survival. (b) OS of 39 patients with *JAK2* allele burden higher than 75%. Patients with *ASXL1* mutation did not have significantly different OS from those without *ASXL1* mutation.

**Supplemental Figure 6. Kaplan-Meier survival curves of 122 PMF patients stratified by the statuses of *ASXL1* and *JAK2* mutations, and *JAK2* mutant allele burden lower or higher than 75% if *JAK2* was mutated.**

Patients with concurrent *ASXL1* mutation and low *JAK2*V617F allele burden had the shortest survival among the total cohort.

**Supplemental Figure 7. Kaplan-Meier survival curves of *JAK2*-mutated PMF patients with allele burden lower or higher than 50% stratified by the status of *ASXL1* mutation.**

(a) OS of 26 patients with *JAK2* allele burden lower than 50%. Patients with *ASXL1* mutation had significantly shortened survival. (b) OS of 53 patients with *JAK2* allele burden higher than 50%. Patients with *ASXL1* mutation did not had significantly different OS from those without *ASXL1* mutation.

**Supplemental Figure 1. Mutational landscape of 122 PMF patients.**


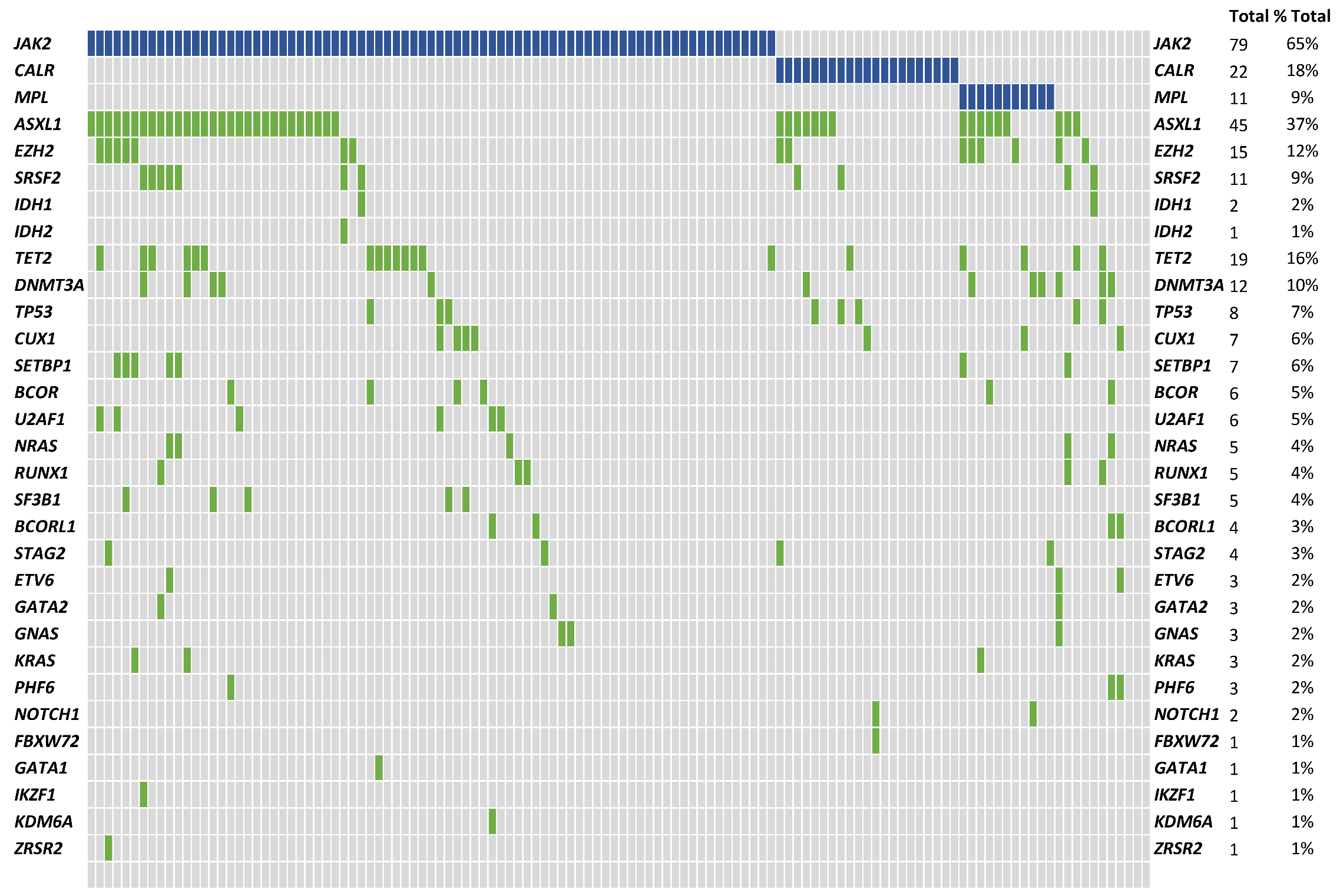


**Supplemental Figure 2.**

| a | b |
| --- | --- |
| 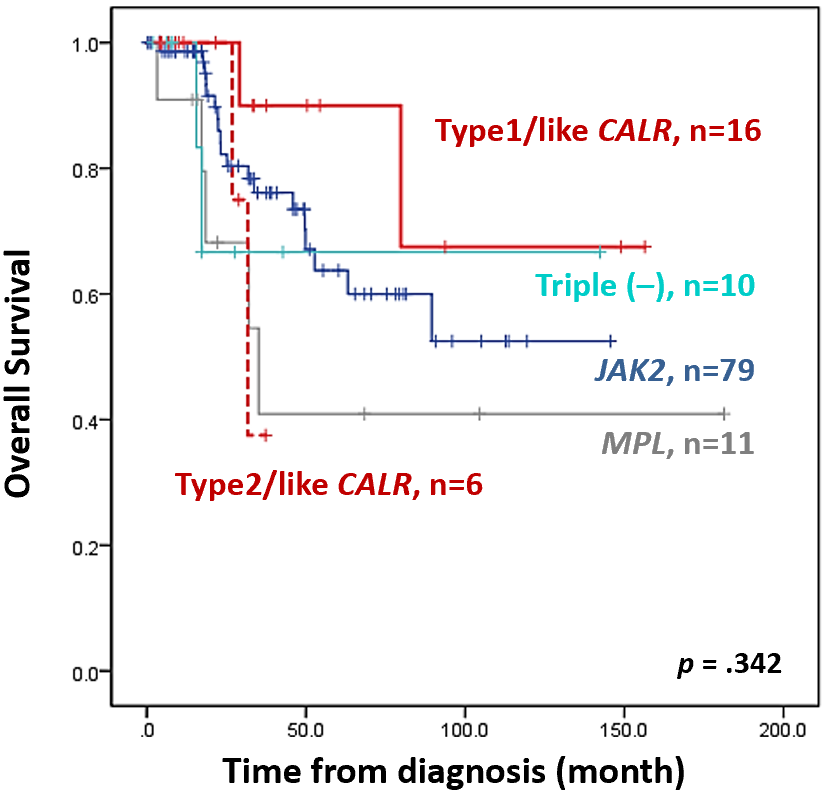 | 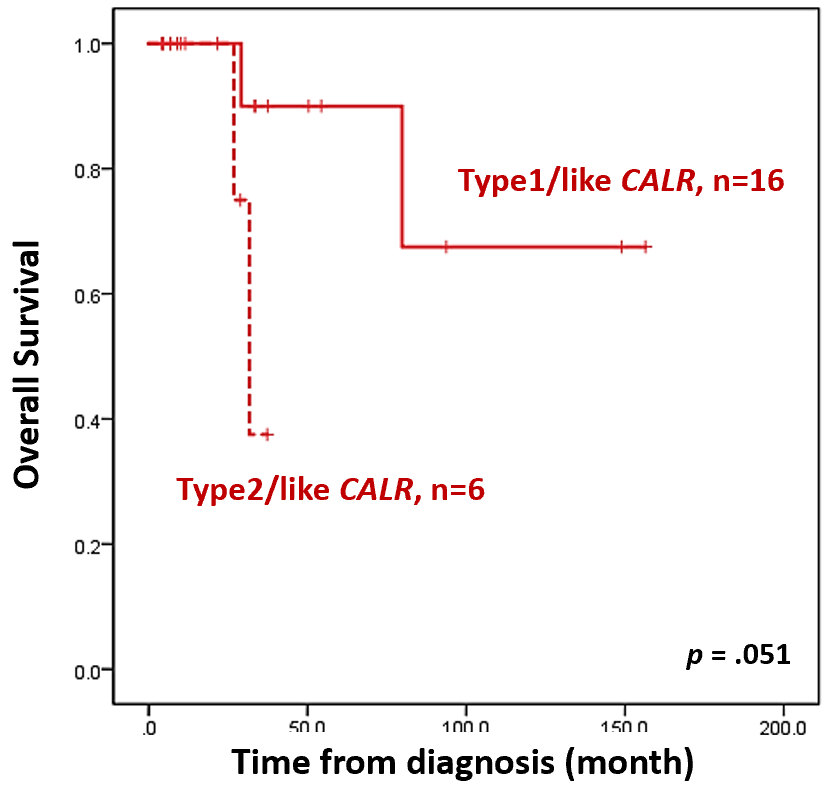 |

**Supplemental Figure 3.**

| a | c | e |
| --- | --- | --- |
| 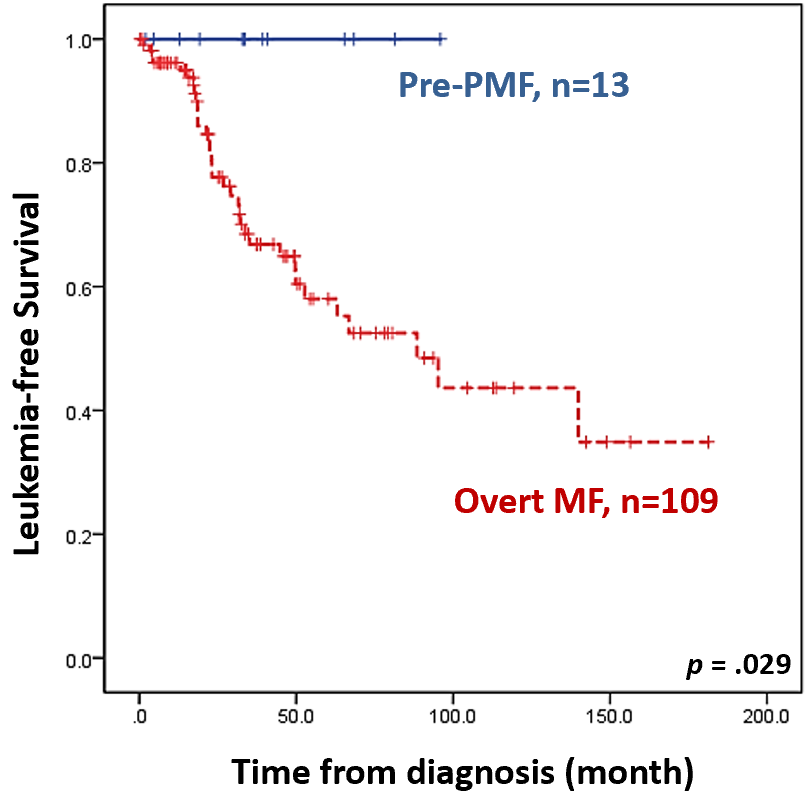 | 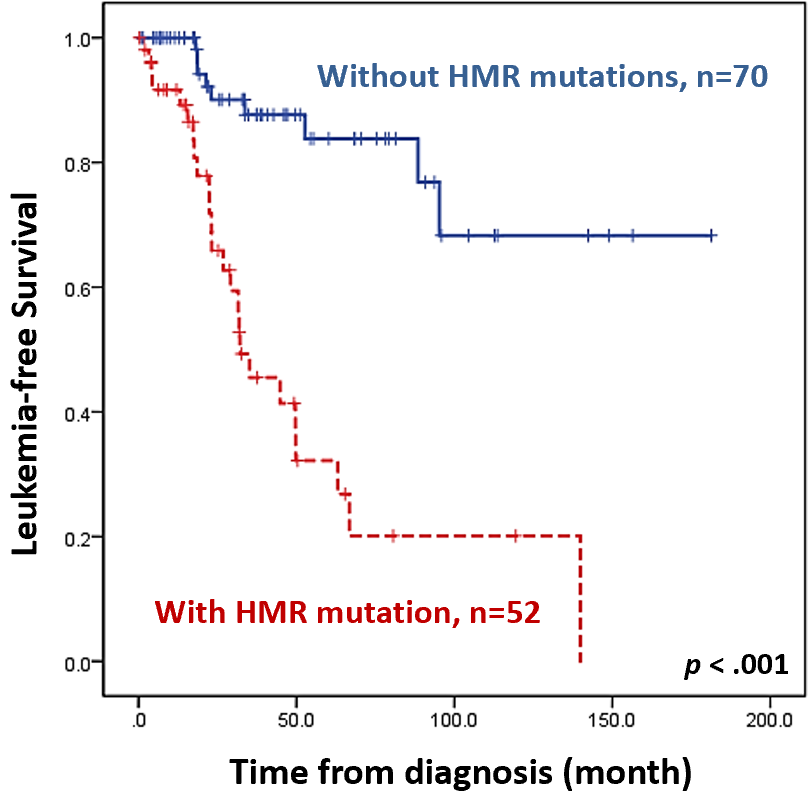 | 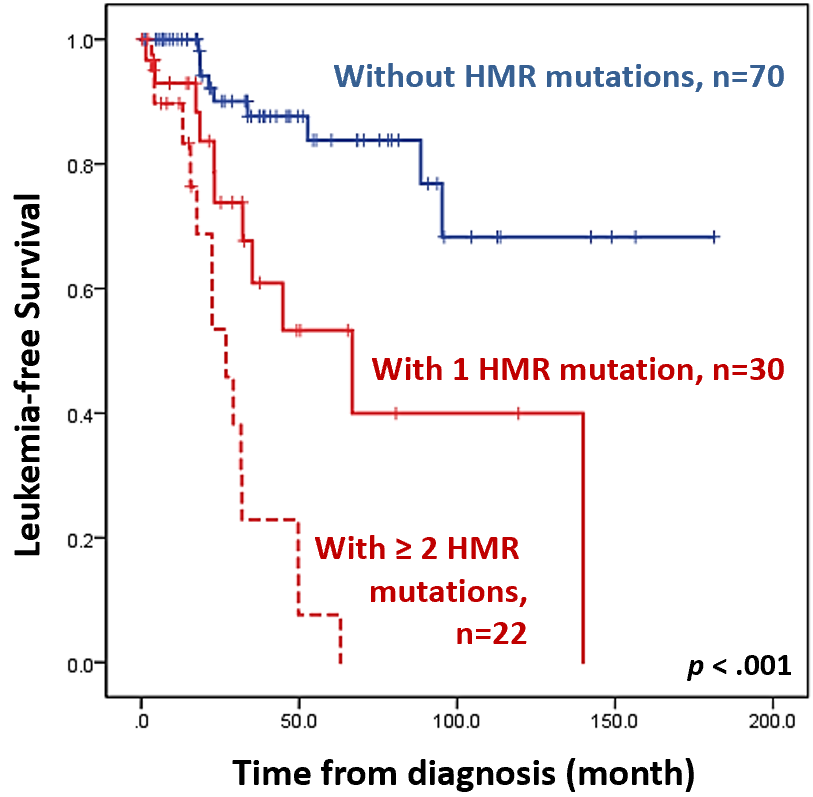 |
| b | d | f |
| 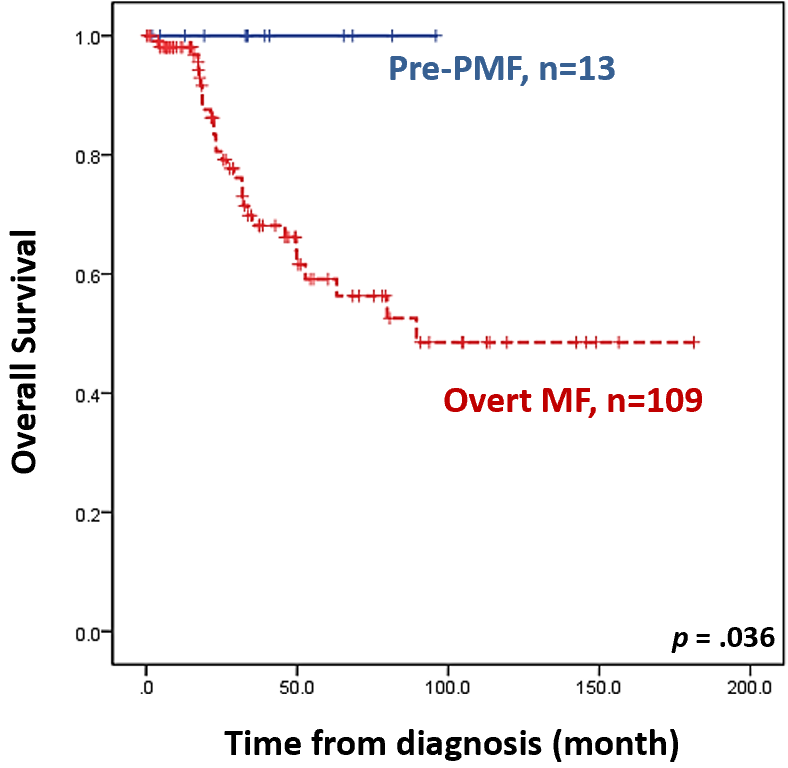 | 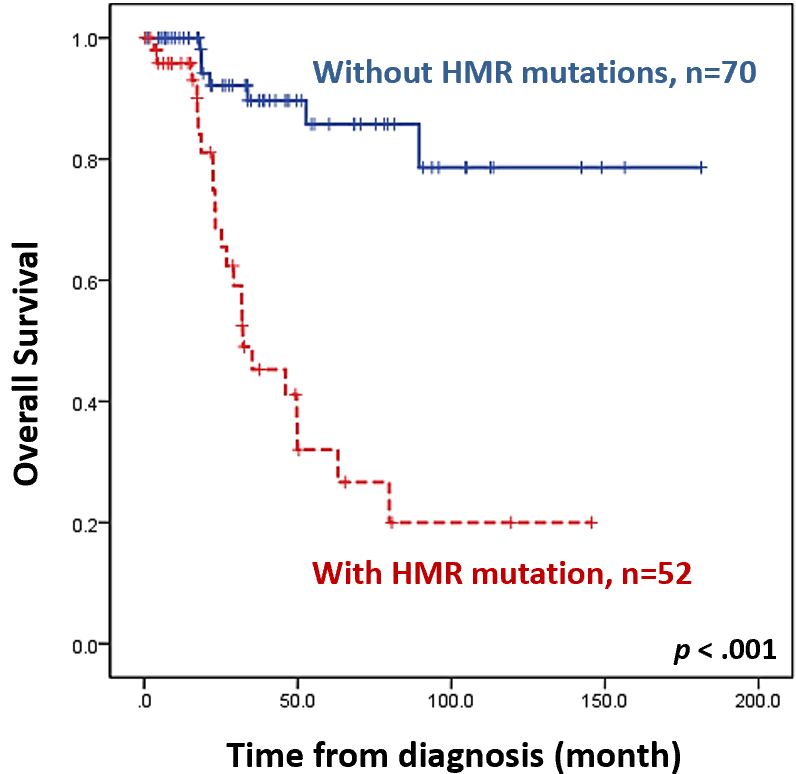 | 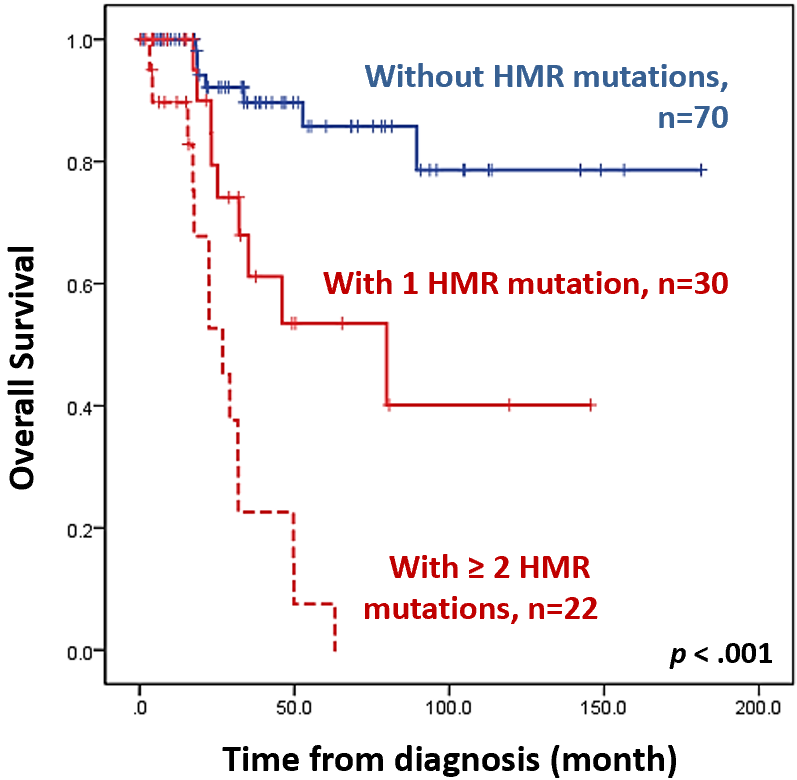 |

**Supplemental Figure 4.**

| a | 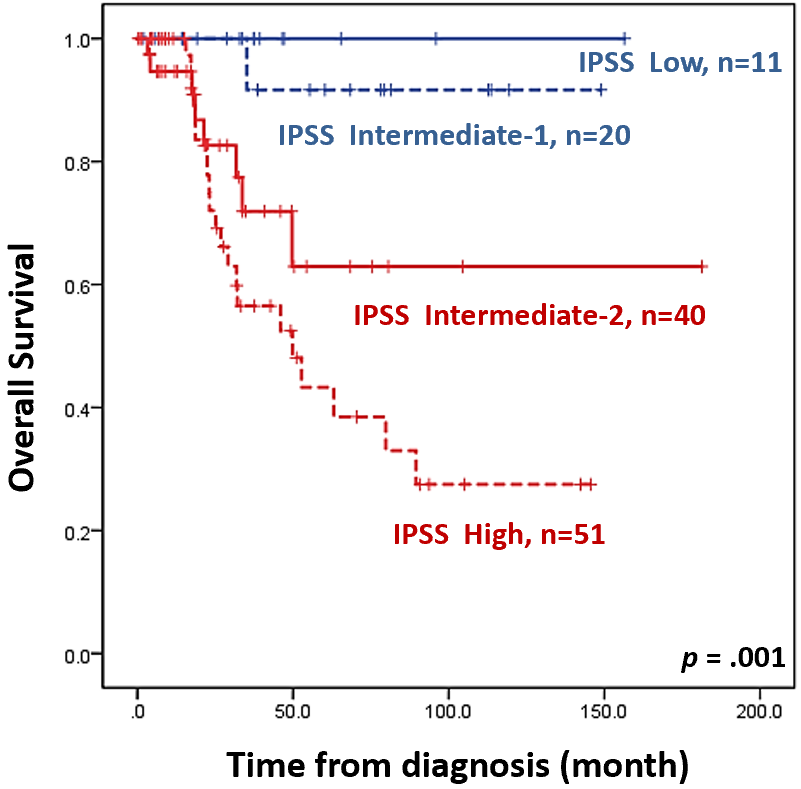 | b | 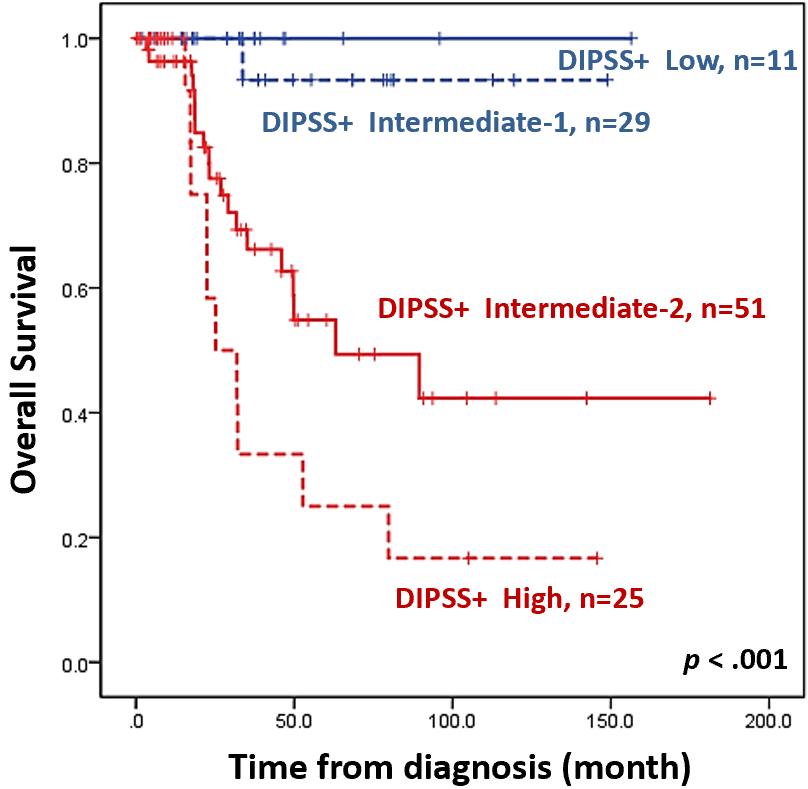 |
| --- | --- | --- | --- |
| c | 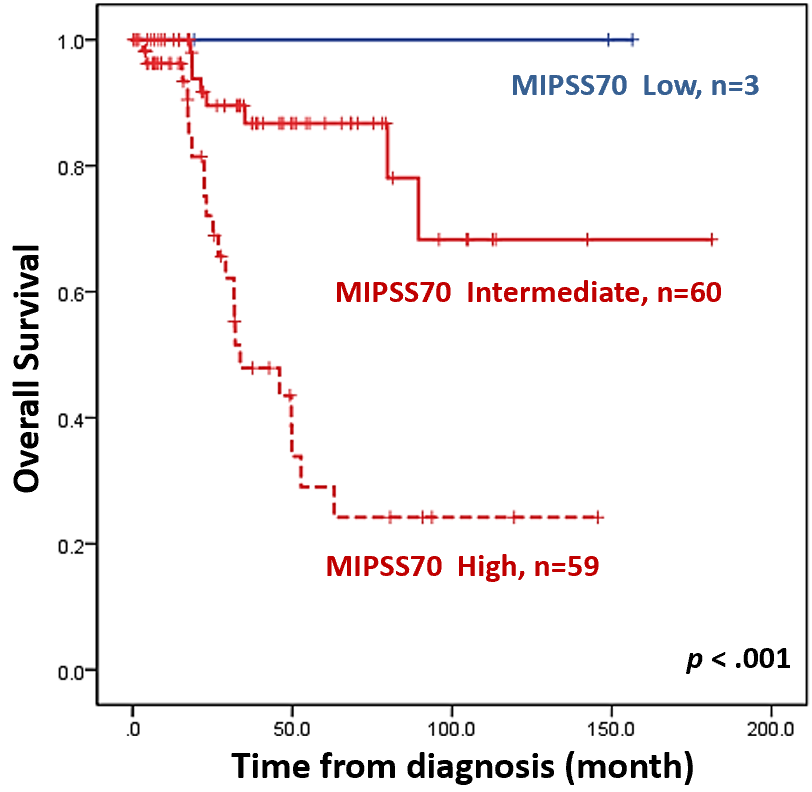 | d | 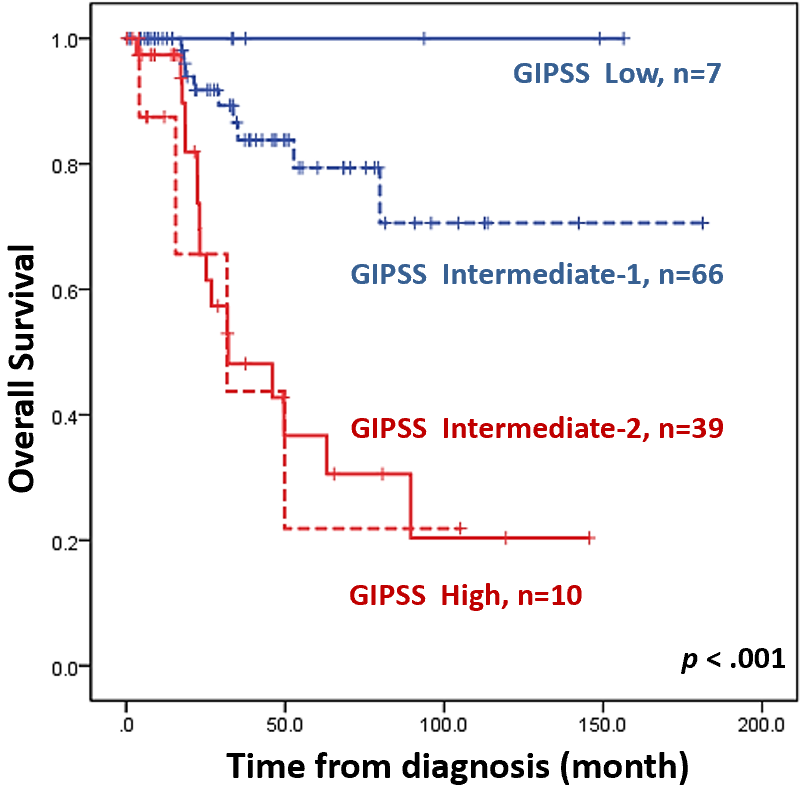 |

**Supplemental Figure 5.**

| a | b |
| --- | --- |
| 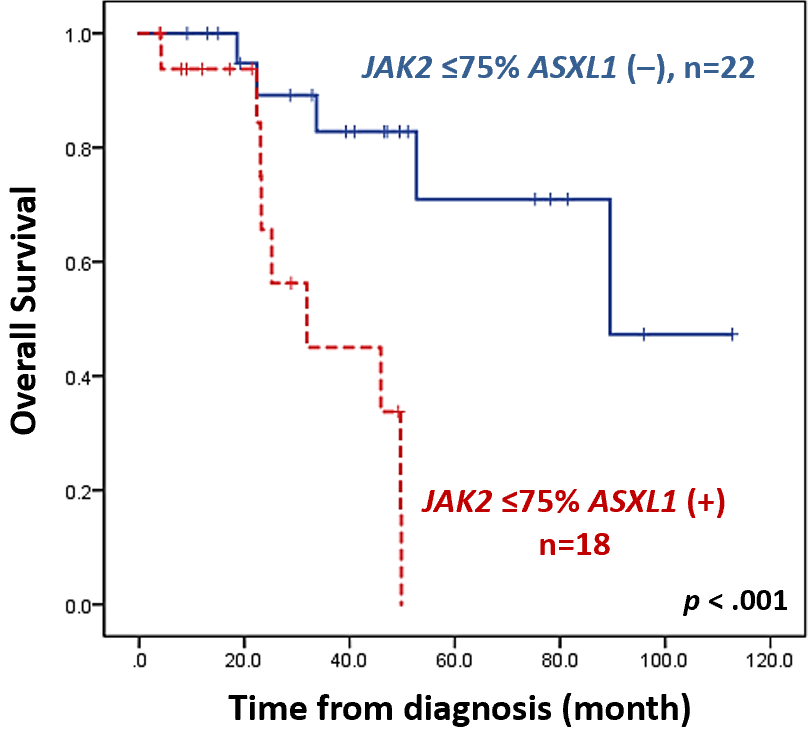 | 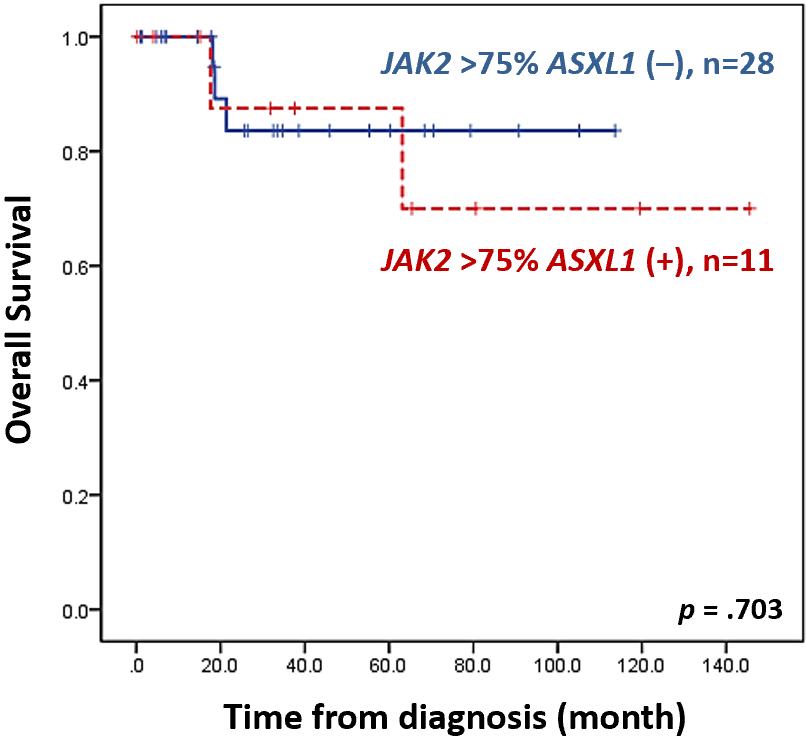 |

**Supplemental Figure 6.**

|  |
| --- |
| 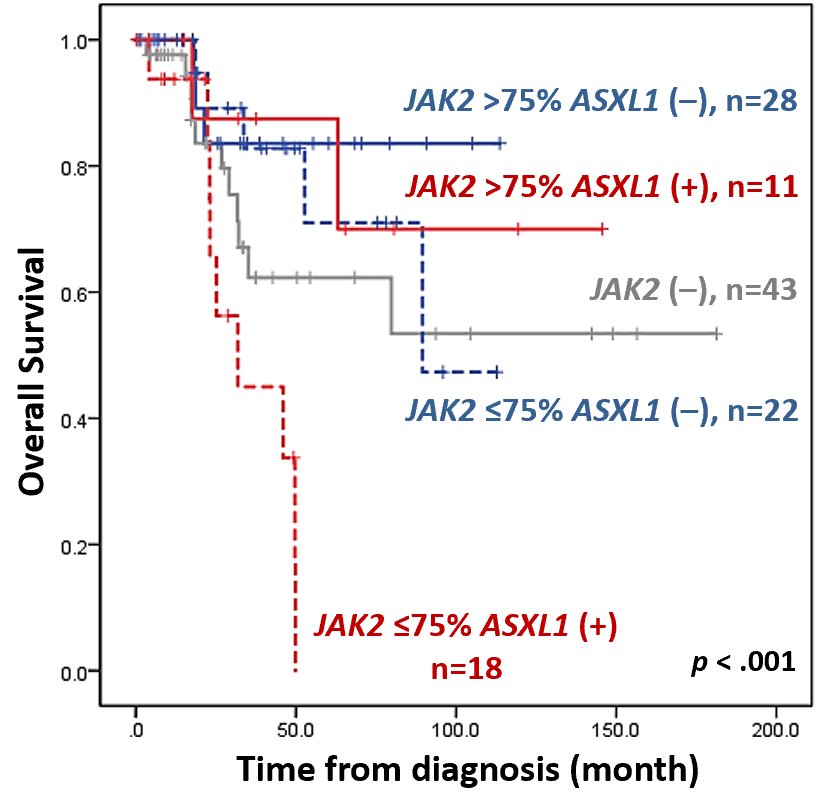 |

**Supplemental Figure 7.**

| a | b |
| --- | --- |
| 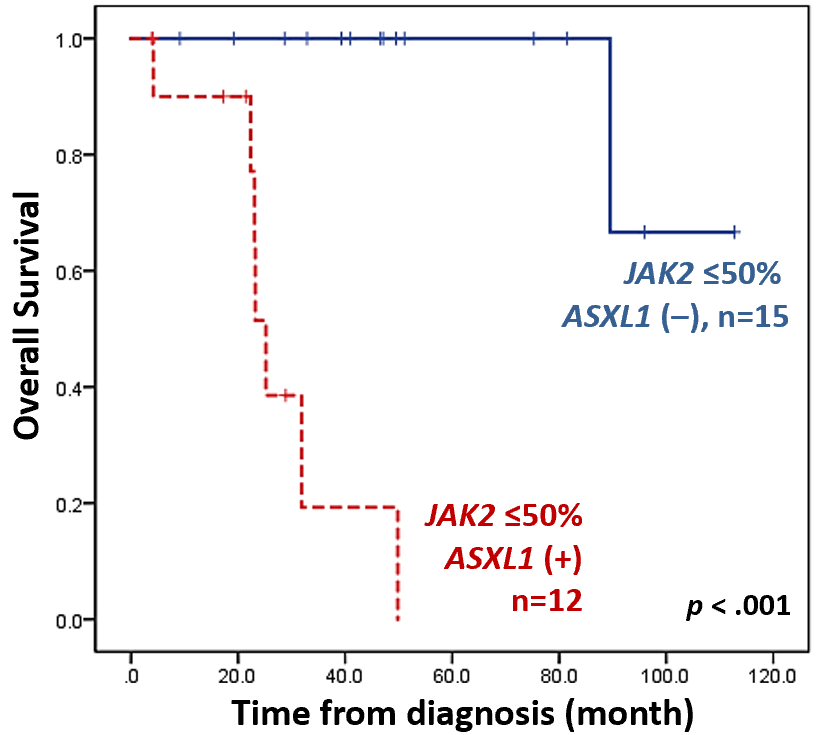 | 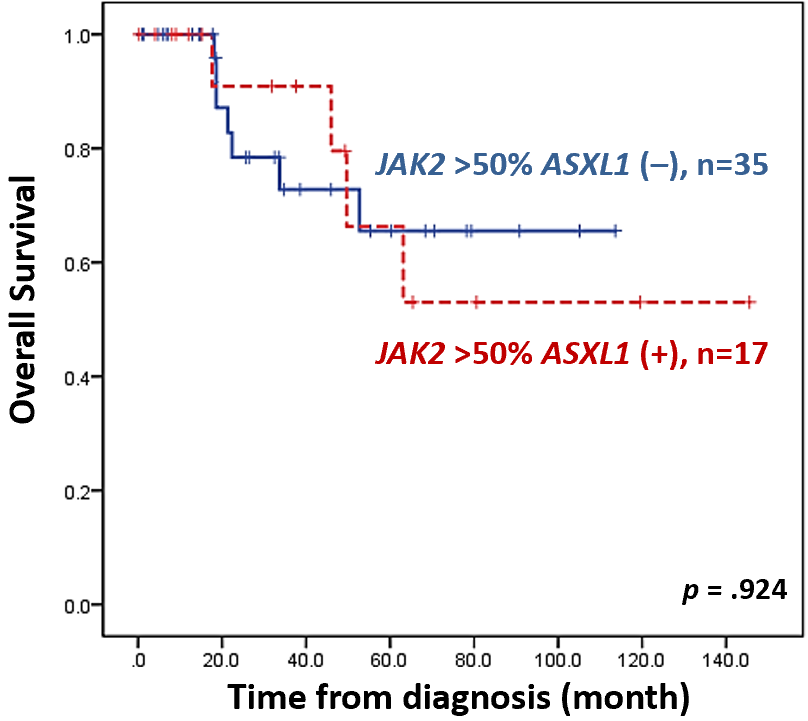 |
